# An optimized protocol with a stepwise approach to identify specific nuclear receptor ligands from cultured mammalian cells

**DOI:** 10.1016/j.xpro.2021.100658

**Published:** 2021-07-07

**Authors:** Alexandre Berthier, Bart Staels, Philippe Lefebvre

**Affiliations:** 1 Univ. Lille, Inserm, CHU Lille, Institut Pasteur de Lille, U1011-EGID, 59000 Lille, France

**Keywords:** Cell Biology, Cell-based Assays, High Throughput Screening, Molecular Biology

## Abstract

Here, we describe an optimized protocol to identify specific nuclear receptor ligands. First, to rule out any compound interference with luciferase activity per se, we describe an *in vitro* assay assessing potential inhibition or activation of luciferase enzymatic activity. Second, to comply with EMA and FDA guidelines to mitigate drug-drug interactions, we detail assays assessing constitutive androstane receptor (CAR) and pregnane X receptor (PXR) activation ability. Finally, to minimize off-target detection effects, we describe the use of mammalian one- (or two-) hybrid systems.

For complete details on the use and execution of this protocol, please refer to Hering et al. (2018).

## Highlights

• Potential nuclear receptor ligands may directly interfere with luciferase activity
• Potential NR ligands frequently activate CAR and PXR
• Potential NR ligands may activate signaling pathways controlling CAR activity
• Off-target effects may be minimized using mammalian 1- or 2-hybrid assays

## Graphical abstract

**Figure:**
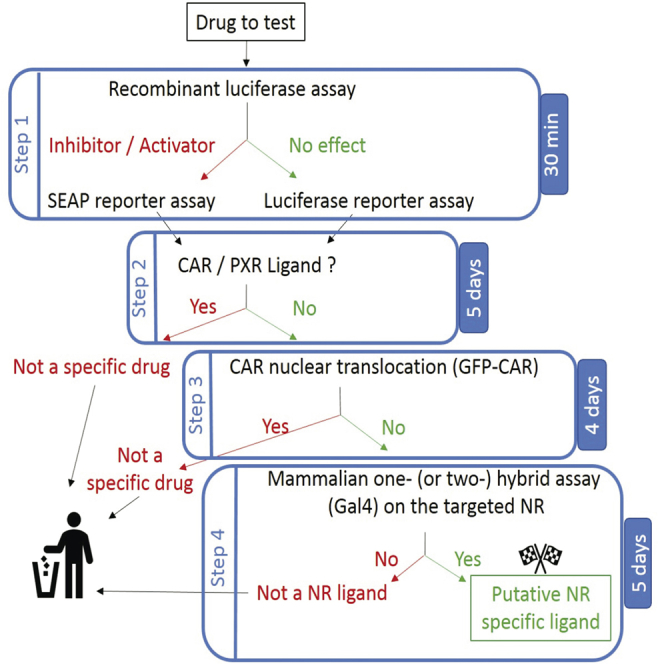

## Teaser

Here, we describe an optimized protocol to identify specific nuclear receptor ligands. First, to rule out any compound interference with luciferase activity per se, we describe an *in vitro* assay assessing potential inhibition or activation of luciferase enzymatic activity. Second, to comply with EMA and FDA guidelines to mitigate drug-drug interactions, we detail assays assessing CAR and PXR activation ability. Finally, to minimize off-target detection effects, we describe the use of mammalian one- (or two-) hybrid systems.

## Before you begin

The protocol below describes the specific steps when using the human hepatocellular carcinoma HepG2 cell line as a model system, but has also been applied to IHH (immortalized human hepatocyte) and HEK293 (human embryonic kidney) cell lines. This protocol requires a luminometer system and an epifluorescence microscope.

### Preparation of solutions for the in vitro recombinant luciferase assay

**Timing: 15 min**

1. Luciferase assay solution [LAS] is prepared as described below and the pH is check and adjust to 7.2 if needed. The LAS solution can be stored for 4 weeks at 4°C.

**Table:** 

Reagent	Final concentration	Stock concentration	Amount
KCl	50 mM	2 M	2.5 mL
BSA	1 mg/mL	N/A	100 mg
Tween-20	0.01%	1%	1 mL
ultra-pure water	N/A	N/A	96.5 mL

2. Prepare luciferase inhibitor II solution (2 mM in DMSO), aliquot and store protected from light at −20°C (solution can be store up to 1 year at −20°C).

**CRITICAL:** The LAS solution can be stored for several weeks at 4°C but must be at 20°C–22°C for the assay**.**

### Preparation of solution and material for the all transcriptional assays

**Timing: 2 days**

3. Charcoal-dextran stripped fetal calf serum (FCS) (troubleshooting 1)
  a. Prepare a suspension of 5% activated charcoal in 500 mL ultra-pure water.
  b. Centrifuge at 4,000 *g* for 5 min and wash 3 times the pellet with distillated water
  c. Centrifuge at 4,000 *g* for 5 min, discard water.
  d. Add 2.5 g dextran and 500 mL FCS and incubate 16 h at 4°C under agitation.
  e. Centrifuge at 4,000 *g* for 20 min at 22°C
  f. Sterilize the solution by filtering it twice on 0.22 μm filters.
  g. Inactivate complement proteins by heating the charcoal-dextran stripped FCS (CD-FCS) at 56°C for 45 min.
  h. Aliquot (50 mL) and store at −20°C
4. Cell growth, transfected cells seeding and cell treatment media (see Tables below for composition).
  a. Cell growth (CG) medium contains 10% FCS and is used for cell culture, seeding and transfection.
  b. Transfected cell seeding (TCS) medium contains 10% of CD-FCS and is used to seed transfected cells.
  c. Cell treatment (CT) medium contains 1% of CD-FCS and is used for drug testing.

**Table:** 

	Stock concentration	Final concentration	Amount
CG medium			
MEM	N/A	1**×**	500 mL
FCS	N/A	10% (v/v)	50 mL
Sodium Pyruvate	100**×**	1 mM (1**×**)	5 mL
Penicillin / Streptomycin	100**×**	100 units/mL penicillin and 100 μg/mL streptomycin (1**×**)	5 mL
NEAA	100**×**	1**×**	5 mL

The CG medium can be store for 4 weeks at 4°C.

**Table:** 

	Stock concentration	Final concentration	Amount
TCS medium			
DMEM w/o phenol red	N/A	1**×**	500 mL
CD-FCS	N/A	10% (v/v)	50 mL
Penicillin/Streptomycin	100**×**	100 units/mL penicillin and 100 μg/mL streptomycin (1**×**)	5 mL
Glutamine	100**×**	2 mM (1**×**)	5 mL

The TCS medium can be store for 4 weeks at 4°C.

**Table:** 

	Stock concentration	Final concentration	Amount
CT medium			
DMEM w/o phenol red	N/A	1**×**	500 mL
CD-FCS	N/A	1% (v/v)	5 mL
Penicillin/Streptomycin	100**×**	100 units/mL penicillin and 100 μg/mL streptomycin (1**×**)	5 mL
Glutamine	100**×**	2 mM (1**×**)	5 mL

The CT medium can be store for 4 weeks at 4°C.

**CRITICAL:** Phenol red blunts CAR and PXR transcriptional activity (Kuncharoenwirat et al., 2020) and impacts also on the transcriptional activity of some other NRs such as the estrogen receptor (Rajendran et al., 1987).

### Preparation of solutions and materials for the GFP-CAR nuclear translocation assay

**Timing: 2 days**

5. Prepare charcoal-dextran stripped fetal calf serum (FCS) and media as described above.
6. Cool down sterile 1 mL pipette tips and 2 mL cryo-tubes for 16 h at −20°C.
7. Thaw Matrigel for 16 h at 4°C.
8. Make 1.2 mL aliquots of Matrigel into cooled 2 mL cryo-tubes using cooled pipette tips and store them at −20°C.
9. Cool down (−20°C) 5 mL pipettes and 12-well plates before adding Matrigel.
10. Prepare 4% paraformaldehyde solution in PBS.
  a. Warm 800 mL of PBS at 60°C and add 40 g of paraformaldehyde powder.
  b. Add 1 N NaOH dropwise until the solution is clear.
  c. Cool down solution until 22°C, adjust the volume to 1 L with PBS.
  d. Check the pH and adjust to 7.0 with 3.7% HCl if needed.
  e. Sterilize the solution by filtering on 0.22 μm filters and aliquot. The solution can be sore for 4 weeks at 4°C or 6 months at −20°C.

**CRITICAL:** Paraformaldehyde is toxic and must be prepared and used under a chemical hood.

## Key resources table

**Table:** 

REAGENT or RESOURCE	SOURCE	IDENTIFIER
**Critical commercial assays**		

Dual-Glo® Luciferase Assay System	Promega	Cat# E2940
Great EscAPe™ SEAP Chemiluminescence Kit 2.0	Takara	Cat# 631738
JetPEI®	Polyplus-transfection	Cat# 101-10N

**Chemicals, peptides, and recombinant proteins**		

Rifampicin	Sigma-Aldrich	Cat# R3501
PK11195	Sigma-Aldrich	Cat# C0424
CITCO (6-(4-chlorophenyl)imidazo[2,1-b][1,3]thiazole-5-carbaldehyde-O-(3,4-dichlorobenzyl)oxime)	Sigma-Aldrich	Cat# C6240
Phenobarbital (Gardenal)	Sanofi-Aventis	Cat# 3286209
Gibco™ EGF Recombinant Human Protein	Fischer Scientific	Cat# PHG0315
BD Matrigel Matrix Phenol Red-Free	BD Biosciences	Cat# 356237
QuantiLum® Recombinant Luciferase	Promega	Cat# E170A
Luciferase Inhibitor II	Calbiochem	Cat# 119114
ATP (10 mM)	Cell Signaling Technology	Cat# 9804
Beetle Luciferin, Potassium Salt	Promega	Cat# E1602
Polyoxyethylene-20 (TWEEN 20)	Bio Basic Canada Inc.	Cat# 9005-64-5
Albumin from bovine serum (BSA)	Sigma-Aldrich	Cat# A7030
Dextran, from *Leuconostoc spp.*	Sigma-Aldrich	Cat# 31390
Activated charcoal	Sigma-Aldrich	Cat# C9157
150 mM NaCl solution sterile filtred 0.2 μm	Polyplus-transfection	Cat# 702-50
PBS	Thermo Fischer Scientific	Cat#14190-094
Minimum Essential Medium (MEM )	Thermo Fischer Scientific	Cat# 31095-029
DMEM low glucose, pyruvate, no glutamine, no phenol red	Thermo Fischer Scientific	Cat# 11880036
MEM Non-Essential Amino Acids Solution (NEAA) (100**×**)	Thermo Fischer Scientific	Cat# 11140050
Penicillin/streptomycin	Thermo Fischer Scientific	Cat# 15140-122
Sodium Pyruvate 100 mM (100**×**)	Thermo Fischer Scientific	Cat# 11360-039
Glutamine 200 mM (100**×**)	Thermo Fischer Scientific	Cat# 25030-024
Trypsin-EDTA	Thermo Fischer Scientific	Cat# 25300-054
Fetal Calf Serum (FCS)	Eurobio	Cat# CVFSVF00-01
Fetal Bovine Serum, charcoal-stripped, USDA-approved regions	Thermo Fischer Scientific	Cat# 12676029
Paraformaldehyde	Sigma-Aldrich	Cat# P6148
Hoechst-33258 pentahydrate	Thermo Fischer Scientific	Cat# H3569
Dako Fluorescent Mounting Medium	Dako North America	Cat# S3023

**Experimental models: cell lines**		

Human HepG2 cell line	ATCC	Cat# HB-8065

**Recombinant DNAs**		

p(UAS)_4_-tk-Luc	Lien et al. 2014	N/A
pSG5-GAL4(DBD)-hPXR(LBD)	Lemaire et al. 2006	N/A
pM (Gal4 empty)	Clontech	Cat# PT3119-5
pGAL4-hCAR1	Hering et al. 2018	N/A
p(UAS)_4_-tk-SEAP	This article	N/A
p-RL (CMV) [renilla luciferase normalization vector]	Promega	Cat# E2261
p-EGFP-hCAR1	GeneCopoeia	Cat# EX-A0964-M29
pGal4-Rev-erbα	Hering et al. 2018	N/A
pVP16-NCoR	Hering et al. 2018	N/A

**Other**		

Victor Light luminometer	PerkinElmer	Model 1420 Luminometer counter
Epifluorescence microscope	Leica Microsystems	Model DMI 6000B
Combitips advanced 5 mL	Eppendorf	Cat# 0030089669
Thermo Scientific Menzel X1000 Round Coverslip dia. 12 mm #1.5 (0.16–0.19mm)	Fischer Scientific	Cat# 11846933
6-cm Diameter plates	Falcon	Cat# 353004
6-Well plates	Thermo Fischer Scientific	Cat# 130184
24-Well plates	Thermo Fischer Scientific	Cat# 930186
White 96-well plates	Greiner Bio-One	Cat# 675074

## Step-by-step method details

### In vitro recombinant luciferase assay

**Timing: 30 min**

Some compounds may inhibit luciferase enzyme activity, leading to an artifactual decrease or increase of the bioluminescent reaction in transfected cells. An increased signal detection may be due to the stabilization of the enzyme by the tested compound, causing its accumulation in the cell. Upon addition of a large excess of luciferin to the cell lysate, the inhibitory compound is displaced and this generates a higher than expected bioluminescent signal independently of transcriptional regulation and thus incompatible with RN ligand identification (Thorne et al., 2010). Thus, this first step allows the selection of the appropriate reporter gene assay [Luciferase or Secreted Embryonic Alkaline Phosphatase (SEAP)] for the next steps (CAR/PXR off target evaluation and targeted NR assay).

1. Prepare **working solution:** dilute recombinant luciferase protein at 10 nM (final concentration) into LAS solution supplemented with 10 μM ATP (final concentration, 1: 1,000 dilution).
2. Dilute 1:2, 1:20 and 1:200 luciferase inhibitor II stock solution in DMSO to obtain 100**×** concentrated solutions (1 mM, 100 and 10 μM).
3. Dilute tested compounds in DMSO to obtain 100**×** concentrated solution (from 1 mM to 10 μM for example) (troubleshooting 2).
4. Prepare 100 μM Luciferin solution into LAS solution supplemented by 10 μM ATP. Protect from light until use.
5. Recombinant luciferase activity determination (assay must be performed at least in triplicates for each condition):
  a. In a white 96-well plate, add in this order:
    i. 90 μL working solution
    ii. 1 μL DMSO [reference] or 1 μL 100**×** luciferase inhibitor II solutions (final concentrations 10, 1 and 0.1 μM) [positive controls] or 1 μL 100**×** tested drug solution (final concentration from 10 μM to 100 nM for example)
    iii. 10 μL of 100 μM luciferin solution (prepare in step 4).
  b. Shake the plate (directly in the luminometer if possible) and collect light signal with a luminometer (acquisition time 1 s).

**CRITICAL:** Since luciferin is light-sensitive, the tube containing the luciferin solution must be wrapped in an aluminum foil and the solution must be added to wells just prior reading. If the tested compounds act on luciferase activity, all further experiments must be carried out using the SEAP reporter construct [p(UAS)_4_-tk-SEAP].

### CAR/PXR activation assay

**Timing: 5 days**

This step allows to comply with EMA and FDA recommendations (guideline to mitigate drug-drug interaction highly regulated by CAR and PXR) by determining the ligand-dependent activation of the xenobiotic receptors PXR (Pregnane X Receptor, *NR1I2*) and CAR (Constitutive Androstane Receptor, *NR1I3*) by the tested drugs (Berthier et al., 2012, Hering et al., 2018).

6. Day 1: Seed 10^6^ HepG2 cells (use cells from 3 to 17 passage number) in 6-well plates using CG medium and incubate for 16 h at 37°C in a humidified atmosphere and 5% CO_2_.
7. Day 2: Co-transfect cells with expression vector [pSG5-GAL4(DBD)-hPXR(LBD) or pGAL4-hCAR1 or pM (Gal4 empty control vector)], and the reporter vector(s) [p(UAS)_4_-tk-Luc and pRL (Luciferase) or [p(UAS)_4_-tk-SEAP (SEAP)].
  a. Prepare the transfection mix) (troubleshooting 3):
    i. DNA mix: in 100 μL sterile 150 mM NaCl add 500 ng PXR, CAR or Gal4 empty expression vector, 1 μg of reporter vector (Luciferase or SEAP) and 100 ng of pRL (in case of luciferase assay). Vortex gently and spin down briefly using a bench top centrifuge.
    ii. JetPEI mix: dilute 5 μL JetPEI transfection reagent in 100 μL 150 mM NaCl. Vortex gently and spin down briefly.
    iii. Add JetPEI mix to DNA mix, vortex the solution immediately and spin down briefly using a bench top centrifuge.
    iv. Incubate at 20 to 22°Cfor 20 min.
  b. Replace medium by 2 mL fresh CG medium.
  c. Add the transfection mix to CG medium on cells and incubate for 24 h at 37°C and 5% CO_2_.
8. Day 3: Seed transfected cells into white 96-well plate.
  a. Wash cells with PBS (warmed at 37°C)
  b. Add 200 μL trypsin-EDTA solution and incubate for 10 min at 37°C.
  c. Add 2 mL TCS medium.
  d. Resuspend cells with a Combitips® and distribute 100 μL of cell suspension in each well (∼4.5**×**10^4^ cells per well).
  e. Incubate for 16 h at 37°C in a humidified atmosphere and 5% CO_2_.
9. Day 4: Cell treatment is carried in triplicate using CT medium (prepare 400 μL [for 3wells] for each condition).
  a. For CAR assay:
    i. Characterization of potential CAR inverse agonist properties:
      • Prepare 0.1% (v/v) DMSO (negative control)
      • Prepare each tested drug (final DMSO concentration 0.1% v/v)
    ii. Characterization of CAR agonist properties:
      • Prepare 10 μM PK11195 (reference for blunted-basal activity)
      • Prepare 10 μM PK11195 with 5 μM CITCO (agonist positive control)
      • Prepare 10 μM PK11195 with tested drugs.
  b. For PXR assay:
    i. Prepare 0.1% (v/v) DMSO (negative control).
    ii. Prepare 10 μM Rifampicin (positive control).
    iii. Prepare each tested drug (final DMSO concentration 0.1% v/v).
  c. Wash cells with PBS (37°C) and treat cells for 24 h with 100 μL of prepared solutions.
10. Day 5: Determination of reporter activities using a luminometer plate reader (troubleshooting 4):
  a. For Luciferase assay, use the Dual-Glo® Luciferase Assay System according to the instructions provided by the manufacturer.
  b. For SEAP assay, use the Great EscAPe™ SEAP Chemiluminescence Kit 2.0 according to the instructions provided by the manufacturer. For the signal normalization use the “in lab” protein quantification method.

**CRITICAL:** If more testing conditions are needed, increase the number of wells at day 1 and pool transfected cells before seeding into 96-well plate to have a homogenous transfected cell population. The human isoform hCAR1 is constitutively active in reporter gene assays, thus drugs must be tested alone to detect inverse agonist activity and in combination with the hCAR1 inverse agonist PK11195 in order to blunt its high basal activity and detect agonist activity.

### GFP-CAR nuclear translocation assay

**Timing: 4 Days**

This step allows the identification of drugs able to activate the xenobiotic nuclear receptor CAR independently of direct ligand-receptor interaction, but rather by triggering its nuclear translocation through signal transduction pathway(s) activation (Koike et al., 2007). It requires an epifluorescence microscope (excitation filter 485 ± 20 nm / emission filter 530 ± 25 nm).

11. Day 1: Seed 2.5**×**10^6^ HepG2 cells in 6 cm-diameter plate in CG medium and incubate for 16 h at 37°C in a humidified atmosphere and 5% CO_2_.
12. Day 2: Transfect cells with the peGFP-hCAR1 expression vector.
  a. Prepare the transfection mix (troubleshooting 3):
    i. DNA mix: dilute 5.5 μg peGFP-hCAR1 expression vector in 250 μL sterile 150 mM NaCl. Vortex gently and spin down briefly using a bench top centrifuge.
    ii. JetPEI mix: dilute 11 μL JetPEI transfection reagent in 250 μL 150 mM sterile NaCl. Vortex gently and spin down briefly using a bench top centrifuge.
    iii. Add JetPEI mix to DNA mix, vortex the solution immediately and spin down briefly.
    iv. Incubate the mix at 20°C–22°C for 20 min.
  b. Replace medium by 5 mL fresh CG medium.
  c. Add the transfection mix on the cells and incubate for 24 h at 37°C.
  d. Pre-cool for 16 h 24-well plate and pipette tip box at −20°C
13. Day 3: Seed transfected cells into phenol red-free Matrigel-coated 24-well plate:
  a. Pre-coat plate with phenol red-free Matrigel: (troubleshooting 5)
    i. Place pre-cooled 24-well plate, pipette tip box and 10 mL cold (4°C) phenol red-free DMEM on ice.
    ii. Thaw Matrigel aliquots on ice (2 mL for one full 24-well plate), mix gently and dilute 2 mL in 4 mL cold phenol red-free DMEM.
    iii. Add 200 μL diluted Matrigel in each well and incubate plate at 20°C–22°C for 1h
  b. Split transfected cells.
    i. Wash cells with pre-warmed PBS (37°C)
    ii. Add 500 μL trypsin-EDTA solution and incubate for 10 min at 37°C.
    iii. Add 5 mL TCS medium, resuspend cells with a Combitips®, count cells and dilute in TCS medium to reach 100,000 to 200,000 cells per mL.
    iv. Remove excess Matrigel and wash carefully wells with pre-warmed PBS.
    v. Add 1 mL cell suspension to each well.
    vi. Incubate plate for 24 h at 37°C and 5% CO_2_.
  c. Day 4: Cell treatment and eGFP-hCAR1 subcellular localization determination
    i. Cell treatment is processed in duplicate in CT medium:
      • Prepare 2.5 mL 0.1% (v/v) DMSO (negative control).
      • Prepare 2.5 mL 1 μM CITCO and 2.5 mL 1 mM phenobarbital (positive controls in 0.1% [v/v] DMSO).
      • Prepare 2.5 mL of tested drugs at appropriate concentration with 0.1% (v/v) DMSO final concentration (troubleshooting 2).
      • Wash cells with PBS and add 1 mL of “cell treatment” solutions.
      • Incubate for 2 h at 37°C and 5% CO_2_.
    ii. For GFP-CAR subcellular localization:
      • Wash cells with PBS
      • Fix cells for 15 min with 1 mL 4% paraformaldehyde solution (work under a fume hood).
      • Wash fixed cell twice with 1 mL PBS.
      • Stain nuclei for 15 min with Hoechst-33258 (1:1000 in PBS)
      • Wash cells 3 times with PBS
      • Remove PBS and add 1 droplet of fluorescent mounting medium. Cover with 12 mm diameter circular cover glasses.
      • Visualize GFP and Hoechst signals with an epifluorescent microscope (excitation 485 nm / emission 530 nm for GFP and excitation 352 nm / emission 451 nm for Hoechst). Take 2 to 3 pictures of 3 different fields for each well (troubleshooting 4).

**Figure 1:**
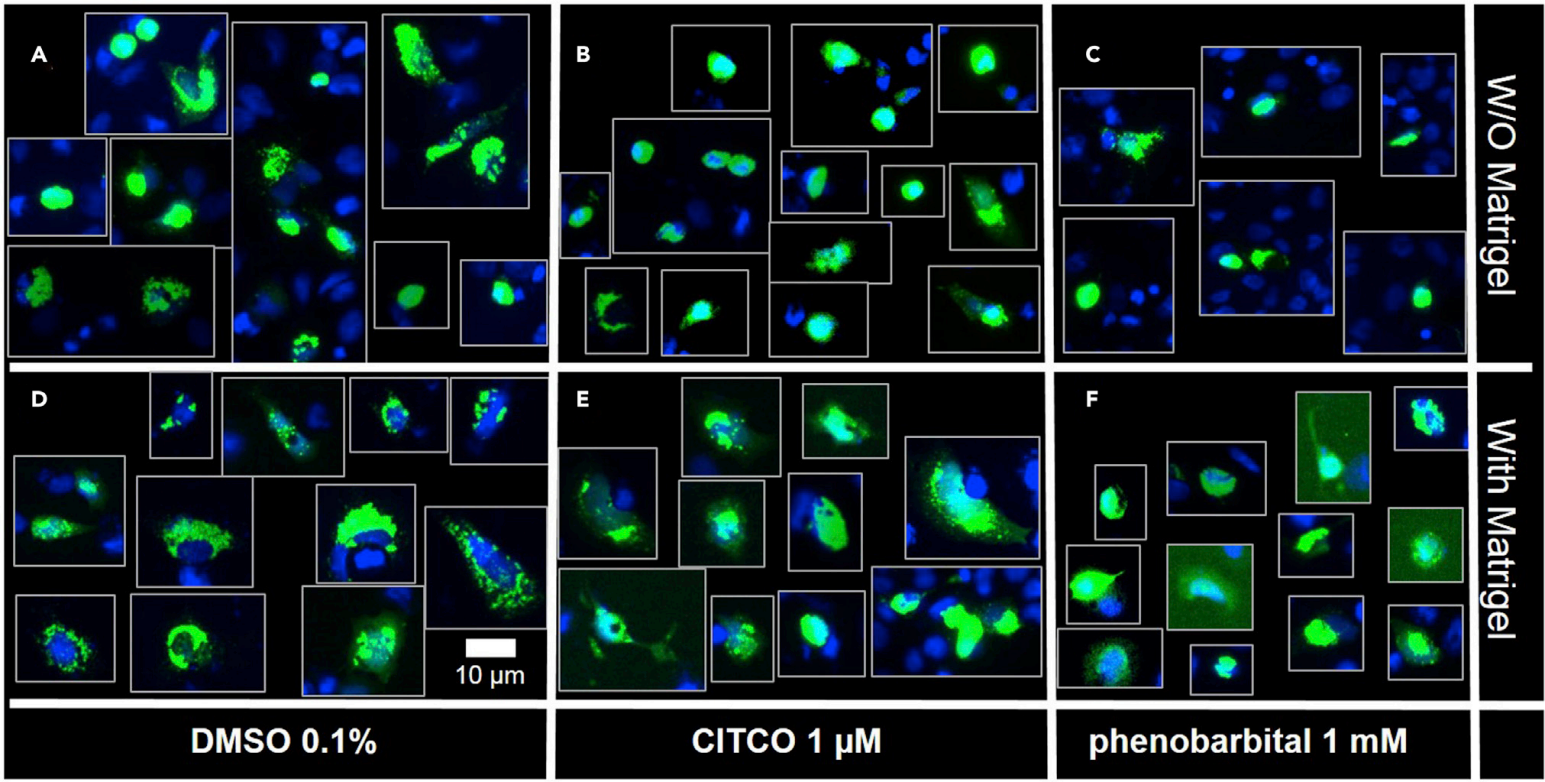
Compound-dependent GFP-hCAR1 nuclear translocation Transfected cells were seeded on plastic (A–C) or on Matrigel-coated wells (D–F). After a 24 h incubation, cells were treated for 2 h with 0.1% DMSO (A and D), 1 μM CITCO (B and E) or 1 mM phenobarbital (C and F). The green signal corresponds to GFP-hCAR1 and the blue signal shows nuclei staining by Hoechst-33258. Scale bar: 10 µM

**CRITICAL:** In contrast to endogenous hepatic CAR, overexpression of eGFP-hCAR1 in cancer cell lines give rise to an artifactual nuclear localization of the exogenous fusion protein (Figure 1 A, B and C). Thereby, in order to study a potential “compound-dependent” nuclear translocation, it is mandatory to first reversibly sequestrate the fusion protein into the cytoplasm. To do so, we developed the above described method based on culture plates pre-coated with Matrigel (Figures 1D–1F). The unliganded-hCAR1 cytosolic-sequestration by the Matrigel is under the control of MEK1/2 kinases activation (for details, see “troubleshooting 5” section of this protocol).

### Targeted nuclear receptor assay: The NR1D1 mammalian two-hybrid assay as an example

**Timing: 5 days**

This step allows the identification of potentially specific ligands for the targeted NR. In this protocol, we describe as an example the procedure allowing the identification of REV-ERBα (NR1D1) ligands using a mammalian two-hybrid system (for full description, see Hering et al., 2018). Briefly, REV-ERBα interacts with NCoR1 to repress target gene expression. The two-hybrid system is based on this interaction, thus cells are co-transfected with pGal4-hREV-ERBα and with pVP16-NCoR. The pVP16-NCoR vector codes for a fusion protein composed by the NR-interaction domain of NCoR1 fused to the transactivation domain of the VP16 virus. The ligand-dependent activation of Gal4-REV-ERBα allows the recruitment of VP16 through its interaction with NCoR and activates reporter gene expression.

14. Day 1: Seed 10^6^ HepG2 cells in 6-well plate using CG medium and incubate for 16 h at 37°C in a humidified atmosphere and 5% CO_2_.
15. Day 2: Co-transfect cell with expression vectors [pGal4-Rev-erbα or pM (Gal4 empty control vector) and pVP16-NCoR], and reporter vector(s) [p(UAS)_4_-tk-Luc and pRL (Luciferase) or [p(UAS)_4_-tk-SEAP (SEAP)].
  a. Prepare the transfection mix (troubleshooting 3):
    i. DNA mix: dilute 500 ng of each expression vector (pGal4-Rev-erbα [or pM] and pVP16-NCoR), 1 μg of reporter vector (Luciferase or SEAP) and 100 ng of pRL (in case of luciferase assay) in 100 μL sterile 150 mM NaCl. Vortex gently and spin down briefly using a bench top centrifuge.
    ii. JetPEI mix: dilute 5 μL JetPEI transfection reagent in 100 μL 150 mM NaCl. Vortex gently and spin down briefly using a bench top centrifuge.
    iii. Add JetPEI mix to DNA mix, vortex the solution immediately and spin down briefly.
    iv. Incubate mix at 20°C–22°C for 20 min.
  b. Replace medium by 2 mL fresh CG medium.
  c. Add the transfection mix on the cells and incubate for 24 h at 37°C.
16. Day 3: Seed transfected cells into white 96-well plate.
  a. Wash cells with pre-warmed PBS (37°C)
  b. Add 200 μL trypsin-EDTA solution and incubate for 10 min at 37°C.
  c. Add 2 mL TCS medium
  d. Resuspend cell with a Combitips® and distribute 100 μL of cell suspension in each well (∼4.5**×**10^4^ cells per well).
17. Day 4: Cell treatment is processed in triplicate using CT medium (prepare 400 μL for each condition).
  a. Prepare 0.1% (v/v) DMSO (negative control).
  b. Prepare 10 μM GSK 4112 (positive control).
  c. Prepare each tested drug (final DMSO concentration 0.1% v/v) alone
  d. Prepare each tested drug in combination with 10 μM GSK 4112 to evaluate the potential antagonist properties of compounds.
  e. Wash cells with pre-warmed PBS (37°C) and treat cells for 24 h with 100 μL of prepared solutions.
18. Day 5: Determination of reporter activities using a luminometer plate reader (troubleshooting 4):
  a. For Luciferase assay use the Dual-Glo® Luciferase Assay System using instructions provided by the manufacturer.
  b. For SEAP assay use the Great EscAPe™ SEAP Chemiluminescence Kit 2.0 using instructions provided by the manufacturer. For signal normalization, use the “in lab” protein quantification method.

***Note:*** Mammalian two-hybrid systems should be preferred when the targeted NR possesses a repressive activity, such as REV-ERBs (NR1D1 and NR1D2). However, mammalian one-hybrid system (with only Gal4-NR and UAS-driven reporter vectors) should be chosen when the NR target has a ligand-dependent transactivation activity.

## Expected outcomes

Classically, NR ligands (agonists, antagonists, inverse agonists) are identified either through their ability to promote the recruitment of transcription comodulators (coactivator or corepressor) in acellular methods such as FRET, or using cellular models such as reporter gene assays using notably luciferase enzymes. Although amenable to high throughput screening procedures, several pitfalls are frequently encountered but rarely taken into account when resorting to these latter assays. They are the ability of potential ligands to (1) interfere directly with luciferase enzymatic activity; or (2) to activate the nuclear xenobiotic receptors CAR (constitutive androstane receptor) and PXR (pregnane X receptor), which exert pleiotropic effects when activated (Daujat-Chavanieu and Gerbal-Chaloin, 2020).

**Figure 2:**
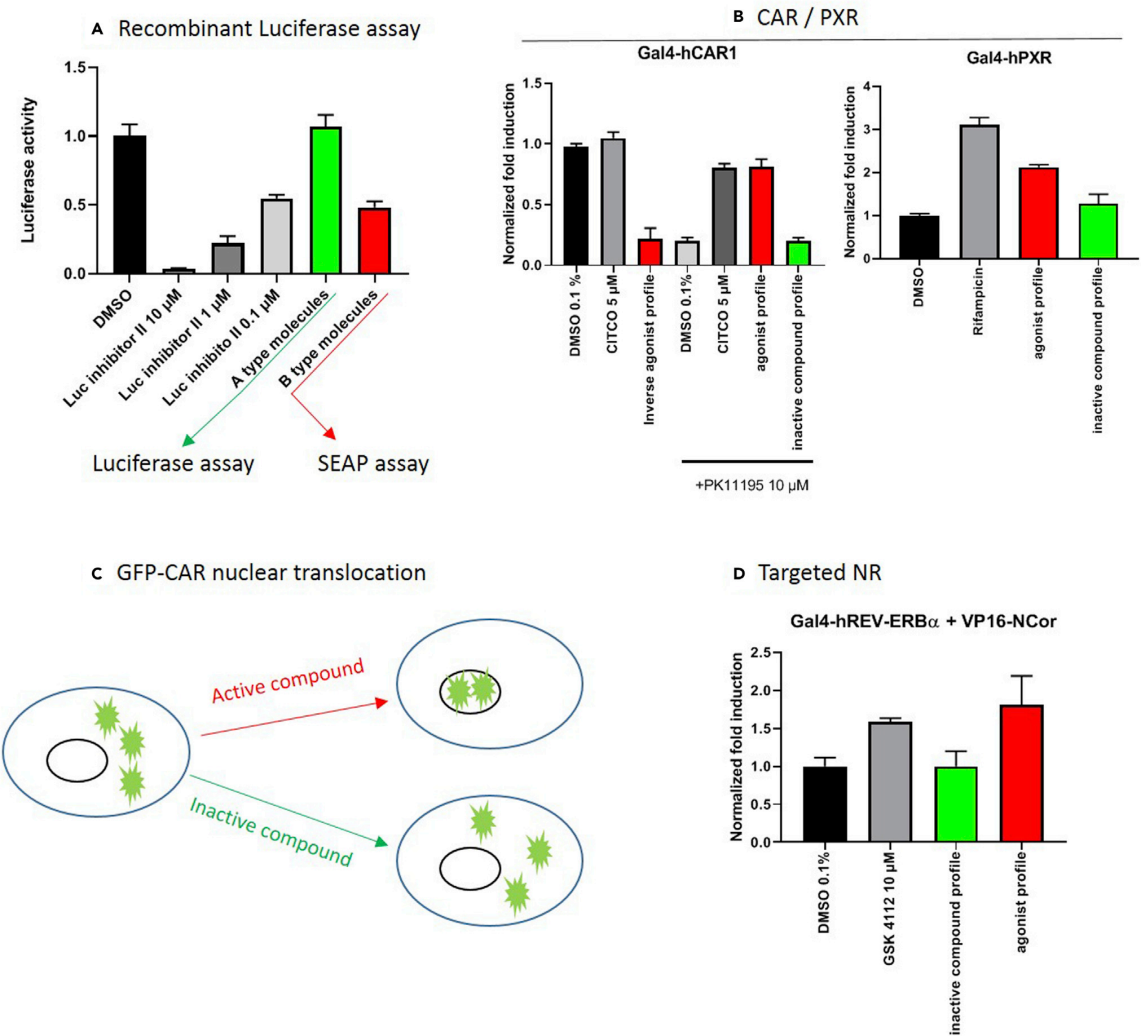
Illustration of activity profiles obtained at each step of the protocol A. Step 1 (recombinant luciferase assay) allows to determine if tested compounds are interfering (red bar) or not (green bar) with luciferase activity per se (“A” type molecules or“B” type molecules respectively). B-type molecules should be tested with an alternate reporter assay such as the SEAP system. B. Step 2 (CAR/PXR) will assess the effect of tested drugs on nuclear xenobiotic receptor activities. Molecules able to modulate their activities (red bar) must be excluded from the initial screening list. C. Step 3 (CAR nuclear translocation) identifies drugs able to modulate the subcellular localization of the constitutively active hCAR1. Active compounds must be discarded. In the last step (step 4, targeted NR [D]), the response of the targeted NR to drugs allows the identification of highly specific hit(s) (red bar). Gray bars are reference compounds in each test.

Here we describe an optimized procedure allowing to by-pass these pitfalls, thus facilitating the identification of more specific NR ligands. This procedure is implemented in 4 steps, first by ruling out any interference of tested compounds with luciferase activity using an in vitro recombinant luciferase assay (Figure 2A). Second, to comply with EMA and FDA recommendations (guideline to mitigate drug-drug interaction highly regulated by CAR and PXR), two sets of assays assessing the ability of compounds to activate CAR and PXR either by direct binding (Figure 2B) or through activation of signal transduction pathways (Figure 2C). Finally, we propose to reduce off-target detection effects by using NR targeted mammalian one- (or two-) hybrid systems (Figure 2D).

## Quantification and statistical analysis

### *In vitro* luciferase assay

The compounds are tested in triplicates, and experiments are run twice. For each molecule, luciferase activity is compared to the control and statistically significant differences are determined using one-way Anova. If the luciferase activity in the presence of 10 μM compound is below 85% of the control, we recommend to process all transcriptional assays using the SEAP reporter system.

### Transcriptional assays

Compounds are tested in triplicates and assays are run 3 times. For each molecule, the normalized reporter activity is compared to the signal obtained first with vehicle (Fold change calculation, [FC]). In a second step, the FC for each compound is compared to the FC induced by the reference molecule. Statistically significant differences are determined using one-way Anova.

### GFP-hCAR1 sub-cellular localization

Hoechst staining allows the detection of DNA/nuclei and is used to visualize the nuclear translocation of the GFP-hCAR1 fusion protein. As the transfection efficiency for HepG2 cells is generally low (≈15%), transfected cells are spread sometimes unevenly in wells. The “in batch” transfection approach allows to uniformly dispatch transfected cells in wells upon splitting. It is mandatory to thoroughly survey each well and take 2–3 pictures in 3 different zones of each well to analyze at least 15 to 20 GFP-stained cells. When the subcellular localization of the fusion protein in one compartment (nuclear or cytoplasmic) is observed for 80% of all GFP-stained cells, it is considered as representative. As this method is purely qualitative, no quantification is needed. When the GFP signal can be localized in the nucleus, and even if a signal can still be detected in the cytoplasm, the tested compound is considered as able to activate the CAR signaling pathway.

## Limitations

In transcriptional assays, FC induced by the reference molecule can be low (from **×**1.5 to 2). In this case, the tested molecule is considered as an agonist when the FC is equal (or higher) to the reference FC. If the reference molecule induces a strong response (FC > 10) the tested molecule is considered as a potential agonist when it induces a FC close to 5 (50% of the reference).

## Troubleshooting

### Problem 1

The removal of hydrophobic and steroid-like molecules from the serum is mandatory to assay NR transcriptional activity. However, this step is time-consuming (2 days) and due to the multiple steps of the procedure, could be a potential source of bacterial contamination (before you begin step 3).

### Potential solution

The “home made” CD-FCS serum can be replaced by a commercial charcoal-stripped FCS depleted from steroid-like molecules and endocrine disruptors. A source for such an alternative is provided in the KRT section.

### Problem 2

The tested compound is not soluble in DMSO (steps 3 and 13-c-i)

### Potential solution

If tested compound is solubilized in a vehicle other than DMSO, the vehicle must also be tested alone (control). The vehicle control (DMSO or else) is essential to provide a formal and quantitative assessment of compounds activity (especially for CAR, PXR and REV-ERBα transcriptional activity assays).

### Problem 3

The JetPEI transfection reagent is compatible with several cell lines (HepG2, IHH and HEK293). The transfection efficiency for HepG2 is compatible for the successful completion of all steps of this protocol. It may however turn out to be low (around 15%–20% in our hand) for the GFP-hCAR subcellular localization assay depending on day-to-day technical variations (step 7, 12, and 15).

### Potential solution

The HepG2 cell line is known to be hard to transfect. However, several transfection reagents are compatible with this cell line [Lipofectamine (Thermo Fischer Scientific) or Fugen HD (Promega)]. To the best of our knowledge, the transfection reagent does not affect the outcome of the various assays.

### Problem 4

No luciferase/SEAP signal is detected (steps 10, 13-c-ii and 18).

### Potential solution

Long term storage of plasmids can be detrimental to transfection efficiency. If the problem persists, all plasmids should be re-amplified and purified.

### Problem 5

The phenol red-free Matrigel pre-coating step is essential to reversibly sequestrate GFP-hCAR1 into the cytoplasm. However, the use of this material is expensive, time-consuming, and requires pre-cooled materials. Matrigel batches quality/efficiency may also vary (step 13).

### Potential solution

**Figure 3:**
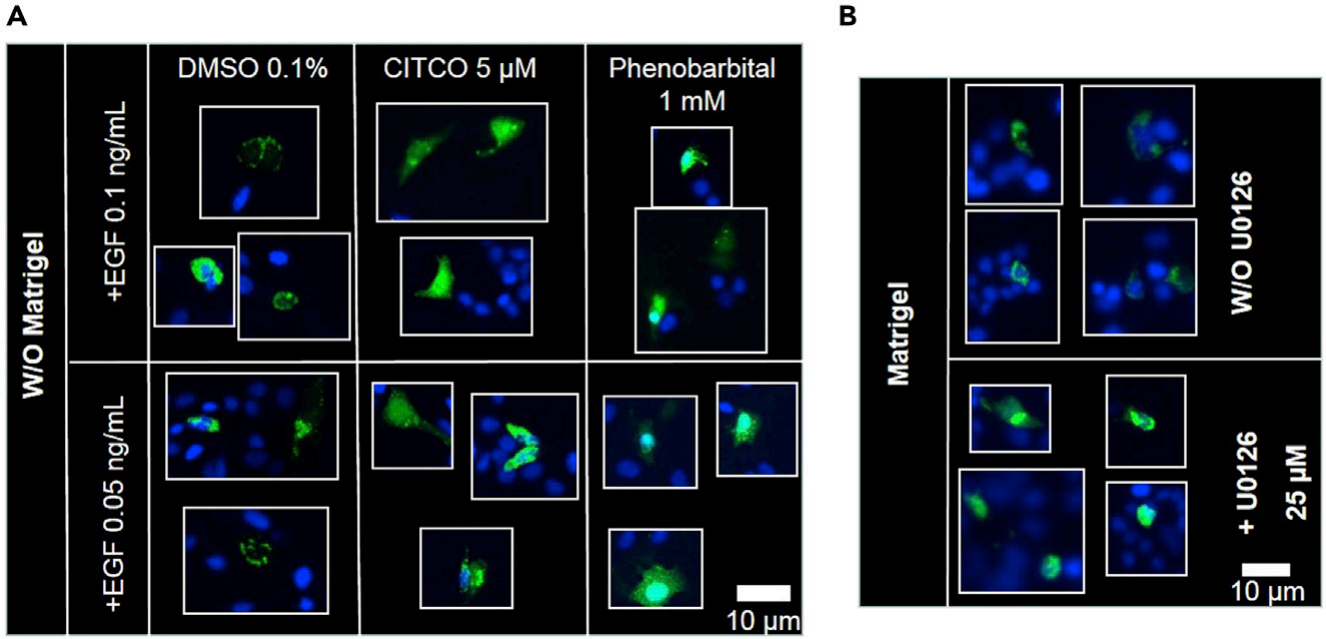
The Matrigel effect on GFP-hCAR1 cytoplasmic sequestration is blunted by U0126 and mimicked by EGF pre-treatment (A) Transfected cells are seeded on plastic, pretreated for 30 min with human recombinant EGF (0.1 or 0.05 ng/mL) and treated for 2 h with 0.1% DMSO, 5 μM CITCO or 1 mM phenobarbital. Scale bar: 10 µM (B) Transfected cells are seeded on Matrigel-coated plate with or without 25 μM U0126. The green signal corresponds to GFP-hCAR1 and the blue signal to nuclei stained by Hoechst-33258. Scale bar: 10 µM

Matrigel reversible cytoplasmic sequestration of GFP CAR can be blunted by the MEK1/2 kinase inhibitor U0126 at 25 μM (Figure 3A, Koike et al., 2007). Of note, Matrigel contains EGF (0.5–1.3 ng/mL depending on the batch). As EGF activates the MEK signaling pathway, the Matrigel pre-coating step can be replaced by a 30 min pre-treatment of transfected cells with (human) recombinant EGF (0.05–0.1 ng/mL) followed by a 2 h co-treatment with drugs (Figure 3B).

## Resource availability

### Lead contact

Further information and requests for resources and reagents should be directed to and will be fulfilled by the lead contact, Philippe Lefebvre (Philippe-claude.lefebvre@inserm.fr).

### Materials availability

Plasmids generated in this study are available upon request to the lead contact.

### Data and code availability

No data or code was generated in this study
